# Editor’s view: Is the brain's perception of ideas an underappreciated human sense?

**DOI:** 10.7189/jogh.14.01002

**Published:** 2024-12-06

**Authors:** Igor Rudan

**Affiliations:** 1 Centre for Global Health, Usher Institute, The University of Edinburgh, UK; 2 Nuffield Department of Primary Care Health Sciences, Oxford University, UK

## Abstract

Humans have developed sensory organs for their ‘major’ senses and specific neuronal receptors for a number of additional senses. Although information from sensors is integrated and processed in the brain, the brain itself has not been proposed as a sensory organ associated with a particular sense – at least not in Western culture or scientific literature. Perception of ideas has many elements of a separate sense, with the brain being a primary sensory organ. I support this notion based on 17 years of experience in the application of the Child Health and Nutrition Research Initiative (CHNRI) method for setting research priorities, which resulted in more than 150 publications involving thousands of experts who prioritised more than 20 000 research ideas. This body of work allows for the generalisation of the following key concepts: defining an idea in the context of the brain's sensory perception; defining the point of origin, sensory qualities, and common types of ideas; considering responses of the body to the brain's sensory exposure to ideas; and defining the key criteria that the brain uses to discriminate between and prioritise ideas. The human brain is continuously being exposed to ideas, which can be self-generated, triggered by information from other sensors, or introduced from the external world. From the brain's sensory perspective, ideas are competing possibilities of purposeful activities that, if followed, would be expected to result in an alternative version of the future. Pursuing ideas tends to drive most of human activity. It requires prioritising between short-, mid-, and long-term investment of energy and time. The brain's sensory role is to continuously assess many competing ideas and prioritise between them based on motivational/emotional (‘attractiveness’), operational/rational (‘feasibility’) and outcome-related perspective (‘impact’). Exposure to new ideas may instigate physiological and psychological responses, ranging from enthusiasm and excitement to feeling a threat or fear. Ideas can be used to mobilise large groups of people whose brains respond with excessive enthusiasm that can sometimes be fanatical. The judgment on the attractiveness, feasibility, and potential impact of ideas is affected by education, experience, and cognitive abilities. This sense may be sharpened through an increased level of expert knowledge and experience. Misinformation and disinformation are dangerous because they affect the brain’s perception of ideas. Impairment of this inherent sense may perhaps contribute to some mental health issues. The proposed view of the brain as the sensor of ideas could lead to many qualitative or quantitative experiments to further explore the properties of this sense in both individuals and populations, establishing ‘the science of ideas’.

Each year during the week of the new Nobel prize winners’ announcements, I tend to recall the quote which is often attributed to one of the former winners, Albert Szent-Györgyi. He wrote: ‘Research is to see what everybody has seen and think what nobody has thought.’ He left that quote in his book ‘Bioenergetics’, published in 1957 [1], likely building on the similar though previously written by Arthur Schopenhauer in 1851 [2], who wrote, in translation from German: ‘Thus, the task is not so much to see what no one yet has seen, but to think what nobody yet has thought about that which everybody sees’ [2,3].

## ‘TO SEE WHAT EVERYBODY HAS SEEN AND THINK WHAT NOBODY HAD THOUGHT’

I have been an active researcher since at least 1993, when I was an undergraduate student of medicine, and have thus taken part in creating new knowledge for more than three decades. I sometimes caught myself wondering whether there has been an occasion in my career where I could say that ‘I have seen what everyone else has seen but thought what no one else thought’. To date, my retrospection could identify only one such instance that might fit this quote well enough, although the circumstances were quite unusual.

To explain them, I need to return to 2007, when I was a consultant for the Child Health and Research Nutrition Initiative (CHNRI) of the Global Forum for Health Research. Supported by the World Bank's grant, I led the development of the ‘CHNRI method’. It was expected to set priorities for health research investments in a systematic, transparent, inclusive, democratic, fair, and replicable way, unlike the previously used approaches based on deliberations by smaller or larger groups of experts. The group which I coordinated over three years has analysed previous attempts to prioritise research ideas [4]. We then developed a conceptual framework that could overcome the key obstacles to a systematic, transparent, democratic, and replicable prioritisation [5], and we explained mechanisms to engage various stakeholders in the process [6]. Finally, we provided detailed step-by-step guidelines for the implementation of this process [7].

The CHNRI method is based on crowdsourcing, a concept introduced to the literature in 2006 – about the same time when we began our work [8]. In the first step, the CHNRI method generates many new ideas solicited from experts in a certain field of health research. Then, it uses the collective opinion of those same experts to assess how each idea satisfies each of the pre-defined criteria, thereby ‘measuring’ which idea is ‘better’ than the other in terms of satisfying the criterion. Thus, the assessment is based on the collective optimism of the larger group of experts. The process results in simple overall scores that range between 0% and 100%. Based on these scores, which can be further modified through input from external stakeholders, many ideas can be generated, assessed, ranked, and then prioritised [7,9].

The CHNRI method is therefore based on crowdsourcing, through which many research ideas are proposed and then scored against pre-defined priority-setting criteria. The informed opinions from a larger number of experts can expose the strengths, weaknesses, and relative ranking of each proposed research idea to research funders and policymakers [7,9]. Since its introduction in 2007-08, the CHNRI method has been used in more than 150 publications led by multilateral organisations (e.g. World Health Organization (WHO)), national governments (e.g. China, India, South Africa, Iran, and many others), and funders (e.g. The Bill and Melinda Gates Foundation, National Institute for Health Research UK) to set research priorities in many areas of both health research and beyond [9,10].

The method is simple and very flexible; with minor modifications, it can be applied to prioritise research ideas in any area of science, which helped it gain popularity quite quickly. Between 2007 and 2015, it already became the most widely used process for setting research priorities, surpassing the Delphi method and other previously used approaches [11]. The many examples of published exercises kept introducing innovations and improvements to the originally proposed method. In 2016, the strategies for engaging funders [12], researchers [13], and other stakeholders [14] were revised and improved, and key conceptual advances were expanded and further clarified [15].

In response to common questions from the reviewers of the papers based on the CHNRI method, I led several additional analyses of previously conducted CHNRI data sets. They provided novel and, to the best of my knowledge, quite original insights into quantitative properties of human collective knowledge [16] and opinion [17]. They showed that very stable results can be obtained with reasonably small sample sizes of engaged experts: after engaging about 45, the results become difficult to change by adding any number of additional scorers. In 2017, I led the review of the first 50 published examples of implementation [10]. It demonstrated natural progress and growth in variants introduced to the CHNRI process that had been evolving in response to increasingly different needs and diverse applications.

Over the past 17 years since the first publication of the CHNRI method, I was invited as a consultant by many groups that used the method to inform priority-setting processes in their area of health research. Usually, all groups were able to identify the experts who could provide the best ideas to tackle any given problem. They were usually either the most productive scientists in the previous period or most highly cited ones, or both. Their initial task – generating hundreds of new research ideas from those experts’ input – is rarely difficult. I am typically invited by the groups that manage the exercise to assist with the second task – ‘consolidating’ several hundred ideas into the final list, which is then sent back to all experts for scoring. At that stage, duplicate ideas are removed and similar ones merged into a single research question.

The process of checking through all the proposed ‘raw ideas’ usually takes place in a meeting room. The ‘management group’, typically about a dozen experts, sit around the elongated table, with four to six of them at each side. They look at a screen on which one research idea after another is being projected for comments. During this process, I usually sit at the top of the table and observe their work.

So, I was often placed in a unique situation: I was able to observe groups of leading experts looking at a series of hundreds of new research ideas projected to them on the screen. They were trying to reword them, merge them with those they found to be similar enough or delete them based on some agreed rules, to produce the final, ‘consolidated’ list of research ideas for scoring that is short, concise, and clear. It was from that unique position that my unexpected insight occurred. While most of the research ideas were presented to them on the screen, they were all sitting still. However, quite rarely, an idea would be displayed on the screen that would cause all their heads to raise and their bodies to straighten upright. From my perspective, they all seemed as if they collectively ‘woke up’ from a rather dull process; they suddenly became energised and started to vividly discuss an unexpected and interesting, provocative, or controversial new idea. This meant, I thought, that the exposure of their brains to a novel and promising research idea has caused an instant physical response from their bodies, as if their brains acted as ‘sensors’ which were exposed to many competing research ideas. Only rare ideas were ‘sensed’ so that their bodies responded physically – as if the brain’s perception of an idea was a distinct, perhaps underappreciated, human sense. I then also realised that some other group of people, with no knowledge of the subject matter, would be unlikely to have the same reaction. They would not possess enough knowledge to understand why the idea was exciting – but they could, perhaps, be excited by some other ideas.

Excessive stimulation of all the ‘major’ human senses leads to physical responses of the body: the eye’s perception of bright light causes frowning and blinking; the skin’s perception of burning heat causes moving of the hand; the ear’s perception of irritating noise leads to covering our ears or removing the source; the nose’s perception of the bad smell causes us to move and find a fresher air; the tongue’s perception of bad taste leads us to spit rotten food out; and loss of balance will prompt us to quickly adjust our position and prevent the fall. It appears that the brain’s perception of ideas can also lead us to pursue physical activities. We can follow those ideas and explore them further – either as scientists or as human beings.

## POSSIBLE IMPLICATIONS OF THIS INSIGHT

At that point, I began to understand that the prioritisation of health research ideas was merely a special case of a more general process that happens often in our brains: the generation, assessment, and prioritisation of any ideas. That process can address any problem for an individual, a group, or the wider society. The experts on any problem or challenge can be gathered, and then the three steps applied: generating, assessing, and prioritising ideas for any purpose. So, humans can be thought of, generally, as units of life with a limited lifespan, restricted to the surface of their planet. Within the limits of their time and space, they are given an opportunity to generate, assess, and then prioritise ideas about what to do. This process happens at the individual level, but it can also happen at the level of groups (e.g. the CHNRI process) or entire populations (e.g. when leaders are choosing between different policies).

Through their long evolution, humans have developed sensory organs for their six ‘major’ senses. Those senses allow them to form the perception of reality outside of their biological bodies. Humans also developed specific neuronal receptors for several additional, ‘minor’ senses. Although all the information from those sensors is integrated and processed in the brain, the brain itself has not been proposed as a sensory organ associated with a particular sense – at least not in the scientific literature, or in ‘Western’ culture. At that point, I became interested in whether the ‘perception of ideas’ could perhaps fulfil some of the criteria to be considered a human sense, with the brain being a primary sensory organ.

To study and better understand the implications of the human brain being ‘a sensor for ideas’ and how could that notion be practically used, some of the questions that would need to be addressed are: defining an idea in the context of the brain's sensory perception; defining the point of origin, main components, qualities and common types of ideas; considering responses of the body to brain's sensory exposure to ideas; and defining the key criteria that the brain uses to discriminate between ideas.

## UNDERSTANDING THE HUMAN BRAIN’S ‘SENSE OF IDEAS’

Within the context of the entire Universe, humans, as biological units, occupy a remarkably limited space and time. Still, they can develop many different ideas about what to do within their context. The context here is clearly very important, because some ideas may seem ‘good’ in one context, but also ‘terrible’ in the other. So, in order to generate and follow ‘good’ ideas that are likely to improve their condition, humans need to be well-informed about their context. They need to have a proper understanding of their present reality and the future consequences of their actions. This understanding is based on their previous experiences and knowledge. This is one of the reasons why memory is important and why very small children may follow obscure ideas in the absence of previous memories.

### Defining an idea in the context of the brain's sensory perception

As a ‘sensor of ideas’, the human brain is being exposed to new ideas almost continuously. From its sensory perspective, ‘ideas’ are competing possibilities of purposeful activities that, if followed, would be expected to result in an alternative version of the future. This component of invested energy, time, and expectations of the altered future based on an idea, is quite important to note. Pursuing various ideas tends to drive most of human activity. It requires prioritising between short-, mid-, and long-term investment of human energy and time.

I will use some basic examples to explain this concept further. We could ask ourselves – why pursue any ideas, in the first place? To answer that question, we could consider the alternative version of the future – i.e. ‘complete inactivity’. What will happen to a human in the complete absence of any ideas that may stimulate their brain as a ‘sensor’? If a person just lies down on the floor, stares at the ceiling, and does nothing, autonomous control of breathing will still prompt them to breathe. They can also decide to keep their eyes open or closed, based on their idea about which of the states would suit them better. They can even develop the idea to stop breathing. From an evolutionary perspective, that would not be a ‘good’ idea, because it would eliminate their genes from the gene pool within minutes. Therefore, a self-preserving autonomous physiological mechanism will intervene and override that idea, and they will continue breathing to keep their brain oxygenated.

Soon enough, the sense of a full bladder will give them an idea to urinate. However, they may exercise pursuing an alternative idea of not doing this. Sooner or later, the autonomous physiological impulses will become unbearable and override that idea again. This means that humans are equipped with many intrinsic physiological mechanisms that will override their ‘free will’, especially if their ideas clash with the need to preserve the physiological functioning of their body. Soon, they will get hungry, but they could also have an idea to resist that sense, as some people do during ‘hunger strikes’. They will also get thirsty and sleepy. Again, they can pursue ideas to resist thirst and sleep, but those ideas will, again, be overridden by their physiology. Clearly, quite a lot of human physical behaviour is dictated by physiological needs that eventually override a desire to pursue some ideas that would conflict with those needs.

However, in periods of existence when they are awake and do not need to satisfy other physiological urges to maintain the functionality of their bodies, humans can prioritise many different and competing ideas. This will lead them to pursue activities which will then determine their health and well-being, as well as the outcomes in life.

### Defining the point of origin, sensory qualities and common types of ideas

In terms of their point of origin, it appears that the ideas can be self-generated, triggered by the information from other sensors in the human body, or introduced from the external world. An example of a self-generated idea might be one that was conceived ‘while in the shower’. When humans disconnect from external inputs and have time to process their memories and thoughts and plan ahead, then they are in the best position to self-generate ideas. This may be one of the reasons why solitude and isolation may help create original works of art or science. An example of an idea triggered by the information from other sensors could be one where people feel an itch and decide to move and scratch, or feel numbness in their legs following long work in the sitting position and then decide to exercise or take a walk. An example of an idea introduced from the external world is one where the car’s radio warns of the traffic jam miles ahead, leading the person to take a different road home. In this, several new ideas of different routes may be generated, and then assessed and prioritised.

Nowadays, human brains are often exposed to externally introduced ideas through (neuro)marketing, propaganda, or any type of ‘influencing’ that will try to direct them towards some kind of behaviour. This is typically in someone else’s interest – usually financial, but it can also be ideological, or related to political processes such as elections. Social networks have become an additional medium suitable for introducing new ideas to many people simultaneously. Unlike traditional media, which are regulated, social media can spread any type of ideas, including those based on misinformation or deliberate disinformation. In many societies, ideas can even be imposed as the expected modes of activity and behaviour through various laws and under the threat of punishment. It appears that many human day-to-day activities consist of pursuing ideas externally incepted from the others.

Those three main points of origin will simultaneously generate ideas that will compete for prioritisation. They will have some ‘sensory qualities’; to the brain as their sensor, they can be interesting, exciting, inspirational, threatening, scary, uninspiring, boring, and destructive, and could seem dangerous, forbidden, or even criminal.

There is seemingly a very broad, diverse spectrum emerging from these main types of ideas that drive human behaviour: business ideas can generate more resources; research ideas more knowledge; political ideas more justice, equity, or representation; religious ideas more peace and acceptance; investment ideas more financial wealth; and so on.

Within each of those categories, there seems to be an opportunity to be systematic in considering and generating all possible ideas for prioritisation. In the CHNRI method, this was captured by the concept of the ‘four D’s’ – description, delivery, development and discovery [5,7]. Thus, there are four broad categories of ideas on how can the future be changed: ideas on how to describe the existing context; ideas on how to do more within the existing context based on the new information, while using the same energy and time; ideas on how to improve the existing context; and ideas on how to change the existing context by inventing something new. This seems to be a rather broad, but still potentially useful starting point for systematic generation of ideas. Conclusively, humans can pursue ideas based on how to describe, optimise, or improve the context, or invent something new to change it. They do this to give themselves more options and better prospects.

It would be interesting to explore how money is related to the process of generating, assessing, and prioritising ideas. As an example, when prioritising ideas on where and how to live, which occupation to pursue, which car to buy, or where to travel for holiday, the ‘better’ the idea seems to most people, the more demand will be generated – i.e. for the specific neighbourhoods of residence, occupations, types of cars, and holiday destinations, driving up their prices, but likely also improving the outcomes in life. Another example to consider is an instant recognition of an ‘ingenious’ idea, which could be, e.g. a musical idea in the form of a new song that everyone likes. If there is an inherent human ability to ‘sense’ ingenious ideas and admire them, perhaps this mechanism could push the collective intelligence towards increased values within the human species. However, history also gives all too many examples where terrible ideas were recognised, admired, and followed with passion, leading to wars, destruction, and immense human suffering.

### Considering the responses of the body to the brain's sensory exposure to ideas

Exposure to new ideas may instigate physiological and psychological responses ranging from enthusiasm and excitement to feeling a threat, fear, or envy. History is full of examples where ideas were used to mobilise large groups of people – the ‘followers’ – whose brains responded to proposed ideas with excessive enthusiasm that could sometimes even be fanatical and radical, to the point of sacrifice of health, sanity, or even life for the idea. Therefore, a variety of the body’s responses to the brain's sensory exposure to ideas should be studied, mapped, and better understood – both at the level of individuals, groups, or entire populations.

It would be particularly interesting to consider whether humans are, to any degree, ‘programmed’ to recognise some ideas and respond to them, both as individuals and as a collective. An example is the remarkably quick and widespread adoption of digital technologies – computers and mobile phones. There is no apparent evolutionary reason why the idea of their adoption should be so attractive to biological entities, given that those technologies never existed in the past. If science could test the hypothesis of whether humans are ‘programmed’ to prioritise some ideas over others, then humanity may indeed have a direction of progress in time through this mechanism. Humans may possibly even be serving a purpose to achieve some goal by tending to collectively prioritise and follow some ideas.

### Defining the key criteria that the brain uses to discriminate between ideas and prioritise them

The brain's sensory role is to continuously assess many competing ideas and prioritise between them. The CHNRI method defined the five ‘template’ criteria that can make a health research idea more or less likely to lead to the reduced burden of disease in an equitable way. In a more general scenario, what criteria would be useful? There seem to be at least three that are reasonably independent from each other.

One such criterion is the ‘attractiveness’ of the idea. It is based mainly on our emotional and motivational perception of the context. Clearly, different things will be ‘attractive’ to different people. Some examples may be buying a nice house, following a girlfriend or a boyfriend to another city, having children, driving an expensive car, becoming really competitive in some sport, travelling the world with a backpack, or becoming wealthy. People will be willing to invest a remarkable amount of their energy and time pursuing some of their ideas because they find them emotionally ‘attractive’.

Another important criterion is ‘feasibility’. It is based mainly on our rational and operational perception of the context. This criterion will examine if the idea is likely to realistically lead the person to the future that they desire and if they have enough energy, time, funding, support and approval from others to pursue it until they reach the desired outcome. This criterion may postpone some ideas that would be too risky to pursue. People whose criterion of ‘feasibility’ is strong are unlikely to overspend on their car, travel, or presents simply because they find those ideas very attractive.

The third criterion is the ‘predicted impact’ on one’s future life. It is based on the human ability to predict future outcomes based on understanding the current context, remembering past experiences, acquiring additional knowledge from other people through conversations and books, and having a ‘vision’ of what the future could bring.

Apart from those three criteria, which can ‘assess’ any person’s idea based on its subjective attractiveness, objective feasibility, and likely impact on their life, there is also a distinct element of time involved. Namely, following some ideas can lead to quick outcomes (e.g. cleaning the apartment), while others can lead to mid-term outcomes (e.g. writing a book) or long-term outcomes (e.g. investing in an equity fund). Clearly, if a person gets trapped in an elevator, notices that someone stole their wallet, feels unexplained chest pain, or suddenly remembers an important deadline that they forgot, then pursuing all previous ideas will need to be postponed. The entirely new set of ideas of how to change their immediate future for better will need to be rapidly considered and prioritised.

Clearly, when the brain ‘juggles’ many ideas that it could pursue, it will prioritise them based on the three criteria, but also time – which means that ‘urgency’ may be seen as yet another basic criterion. Prioritisation will also be based on the understanding of the context in which this prioritisation takes place – which may be accurate or rather misinformed.

## THE IMPORTANT ROLE OF THE CONTEXT AND WHY MISINFORMATION AND DISINFORMATION ARE DANGEROUS

In a context where we notice a car that is heading towards us in our own lane, turning the wheel to the side to avoid a direct head-on collision would seem like a very good and potentially life-saving idea. However, turning the wheel to the side when driving on a clear road with no traffic ahead would seem like a terrible and potentially life-threatening idea. The important conclusion is that the context critically matters to the brain’s perception of ideas and to their prioritising. This is reflected in the CHNRI method, where a lot of effort before the launch of each exercise was focussed on carefully defining the context for prioritisation [5,7]. In the CHNRI process, the element of time, i.e. the urgency of outcomes, is one of the five elements of the context.

I started considering the generalised messages of the CHNRI exercises back in 2015 when I began collecting and reviewing the first 50 conducted exercises [10]. It struck me that the people who manage to change their future towards the desired outcomes – the ‘successful’ ones – will often be those who perceive and understand their own context exceptionally well, as well as the causes and consequences of different actions. This will allow them to make good predictions of the causal relationships between their actions in the present and the outcomes in the future. Therefore, success is associated with knowledge, experience, education, and overall cognitive abilities.

Famously, Warren Buffet was second to none in interviewing the CEOs of the companies to understand the context of any industry of his interest, allowing him to invest in companies that became more valuable in the future within that sector. His superb understanding of the present context allowed him to pursue better ideas for investing than other investors.

I then tried to demonstrate that a better understanding of the context will lead to prioritising better ideas. I conducted a study among undergraduate medical students in 2016 to demonstrate that, in order for the best ideas to come out on top, experts with the best understanding of the context should be generating and assessing ideas. The medical students, as a collective of up to 200 individuals, were nearly flawless in collectively answering the questions that they learned in the previous year of their medical studies; however, they did less well when asked general knowledge questions, and then extremely poorly in predicting answers related to the astronomy, which students in that field would know very well [16]. Therefore, when prioritising ideas on how to solve a certain problem, inviting the most knowledgeable experts into a ‘crowdsourcing’ exercise like the CHNRI would be the right approach. At the individual level, persons with less information about their context will likely prioritise ideas that may not serve them well. Over the course of life, this difference will lead to large inequalities between individuals in their outcomes.

However, this also implies that the era of ‘post-truth’ which we entered and the disinformation that is being deliberately spread may bring about serious issues for society as a whole. My spouse recently remarked that she does not know where to get relevant information anymore and noted that ‘we are no longer being informed, but rather influenced’. If people are systematically disinformed about our shared context, this will lead them to prioritise ideas that may harm their individual and collective futures, and to a context where some questionable ideas might be followed by many people.

Simple examples are that, if the people wrongly believe that things are economically worse than they actually are, they may then vote for a change rather than rewarding a government that has a good track record. Also, if negative propaganda is being maliciously spread against a minority group in the population, then many people may support reducing their rights or prosecuting them, although, in reality, those people may be the least problematic group within society. Disinformation impairs people's capacity to make good decisions and follow good ideas.

## ENVISAGING THE ‘SCIENCE OF IDEAS’

The large experience with the implementation of the CHNRI method to date should be carefully reviewed, so that the changes that were ‘intuitively’ introduced by the many users in response to their specific needs can be understood very well. This will assist in working towards ‘the science of ideas’, in which the process of generating, assessing, and prioritising ideas should be scientific. This could be achieved by developing appropriate guidelines, as is good practice in many other areas of science, which would define standard procedures. They would relate to sampling the experts, handling their potential inherent biases, studying agreement statistics, and assessing the uncertainty of the results by providing confidence intervals – all of which were introduced in one of the most recent CHNRI exercises [18].

Studying the first 50 CHNRI exercises allowed me to conceptualise two exercises that I found surprisingly novel: studying quantitative properties of human collective knowledge [16] and of human collective opinion [17]. I was surprised by the absence of previous research in this area, because it seemed an interesting problem. Those two papers convincingly demonstrated that experts, and not lay people, should be used in priority-setting exercises where many different ideas are compared [16], and that the saturation of human collective opinion happens surprisingly quickly – after about 45 experts are involved in the exercise by providing their ideas and opinions [17]. Beyond that number, the addition of many further experts would be almost entirely unlikely to change the overall scores of the proposed research ideas and their ranks on the list.

Nowadays, once the CHNRI exercise is completed, it becomes possible to compare the ranks of prioritised ideas based on human collective opinion to the results derived from the use of artificial intelligence (e.g. ChatGPT), which stores human collective knowledge. This has also been done for the first time in a recent CHNRI exercise [18]. The results showed surprising similarities, but also interesting differences, which may provide clues on how and why human collective intelligence differs from artificial intelligence.

To develop ‘the science of ideas’, the approach to defining the context will need to be standardised. Also, guidelines for choosing the most informative mutually independent criteria that could discriminate between ‘better’ and ‘worse’ ideas will be required. Further guidelines on generating and formulating ideas to make them easily ‘assessed’ by their attractiveness, feasibility, impact, or any other appropriate criteria, will also be needed. Cluster analysis to check whether there were ‘sub-groups’ of experts within a sample that share a dominant opinion, thus influencing the overall results, would be likewise be useful, so that potential biases could be controlled for.

## POSSIBLE PRACTICAL IMPLICATIONS

Developing the ‘science of ideas’ further and strengthening its rigour and replicability could have several practical implications. Many of the challenging and urgent questions in modern times are highly complex. When difficult societal problems need to be addressed, it is difficult for any person to fully grasp all that may be at stake. This is especially true in the ‘post-truth’ era, when many people choose to believe disinformation on social networks rather than any expert opinion.

Therefore, when making important decisions with long-term, far-reaching consequences for entire nations, the approach based on ‘the science of ideas’ could perhaps be considered by governments: aside from any referendum or popular vote, they could also involve larger groups of independent experts and consider their collective opinion, too. Their inclusion could serve as a counterbalance to the concern that too many people may be misinformed about the question. Therefore, decision-making based on collective expert opinion might find its application in developing new policies, legal frameworks, or policymaking on complex challenges.

The ‘science of ideas’ could also assist governments in entertaining many possible approaches on how to solve contemporary issues. In the private sector, it could be used to advise companies on their strategic priorities based on the ideas provided by the leading managers at different levels of the company. Applications may also be found in prioritising ideas to mitigate the damaging effects of disinformation, preventing violence, instability, and inequity. The crowdsourced, collective opinion of the leading experts could also be of large assistance to fact-checking services. It may reduce the risks of hate speech, strengthen the stability of society, and reduce hate crime. This could be achieved by getting clarity on the collective views and positions of those who are best informed by reducing the opportunity for their individual opinions to be manipulated and taken out of context by those engaged in disinformation.

As the effective and successful prioritisation of many competing ideas is likely affected by personal level of knowledge, experience, education, and cognitive abilities, perhaps it would be possible to generate approaches to sharpen this ‘sense’ using virtual reality tools. They could enable training of decision-making in different contexts, both physical and social, based on the best available information. In mental health, it may be possible to explore whether any known issues may arise when the brain’s sensory role in the ‘perception of ideas’ is impaired. Our other senses can also be impaired, leading to various morbidities. As a purely theoretical example, if the brain’s ‘perception of ideas’ becomes hypersensitive, a person could become over-excited with just about any ideas, generating lots of stimulation and energy in the body, which would lead to behaviour similar to that in the manic disorder. Conversely, hyposensitivity of the brain’s ‘sensory’ role would lead to under-excitement towards any ideas, both those self-generated and imputed from the external world, similar to depressive symptoms. If the brain’s sensory activity towards the perceived ideas fluctuated between over- and under-excitement, this would resemble the symptoms of bipolar disorder. The inability to generate or to prioritise coherent ideas or those that are in touch with the context, or a failure to dismiss ideas that should not be considered, would lead to behaviours similar to some described in schizophrenia. If an overwhelming number of ideas were being generated in a unit of time without the ability to focus on any particular one for long enough or follow them through action, that would have similarities to some symptoms observed in autism spectrum disorder.

The brain’s perception of ideas may also be under hormonal influences. It is well known that teenagers become prone to generating and prioritising rather irrational ideas during puberty, which may even endanger their lives – such as running away from home, stealing cars, driving drunk, or experimenting with drugs. Another period in life under hormonal influences – menopause and andropause – is associated with the symptoms of a ‘mid-life crisis’. Some of those symptoms are consistent with an impaired prioritisation of ideas: drastic career changes, ending relationships, moving homes, buying expensive cars, experimenting with risky behaviours, and/or entering conflicts. A special case that may possibly also relate to hormonal influences on the brain’s perception of ideas is ‘heartache’, during which the same poor choices and obsessive behavioural patterns are repeatedly prioritised over much better ones, even though a person fully realises this, but is unable to change.

## DISCUSSION

This editorial seemed like an appropriate form for the paper reflecting on 17 years of the experience with implementation of the CHNRI method for setting research priorities. Namely, the editors of scientific journals should relate to the proposed concepts because their job includes reading countless submitted research ideas. They then need to prioritise which ones to publish and which to reject. This is why their ‘sense of ideas’ – at least the research ideas – inevitably becomes quite sharp. They also need to predict the future impact of each paper based on its novelty, quality, relevance to the field, the likelihood that it will be widely used and cited over time, and several other relevant criteria, thus expanding humanity’s collective knowledge.

This paper revolves around the concepts of ‘idea’, ‘perception” and ‘sense’, so they should perhaps be reflected upon within the wider context. Many philosophers have considered ‘ideas’ to be a fundamental ontological category of being. The capacity to create and understand the meaning of ideas is considered to be an essential and defining feature of humans. Plato was one of the earliest philosophers to provide a detailed discussion of ideas [19]. Since his time, this topic has attracted the leading minds in philosophy, such as John Locke in the 17th century, David Hume and Immanuel Kant in the 18th century, Arthur Schopenhauer in the 19th century, and Bertrand Russell, Ludwig Wittgenstein, and Karl Popper in the 20th century. However, to the best of my understanding of their work, the closest among them to the concepts that I offer in this paper was Rudolf Steiner [19]. He saw ideas as ‘objects of experience which the mind apprehends, much as the eye apprehends light’. In his work ‘Goethean Science’ in 1883, he declared that ‘Thinking (…) is no more and no less an organ of perception than the eye or ear. Just as the eye perceives colours and the ear sounds, so thinking perceives ideas.’ He also believed that this was the premise upon which Goethe made his natural-scientific observations [19,20]. Interestingly, Steiner also developed a social philosophy that centres on the idea of ‘3-foldness’, stating that the human soul consists of thinking, feeling, and willing, and that this is reflected in the activities of the head, the nervous-circulatory system, and the metabolic-limb system of the physical organism. This has similarities to the three ‘core’ criteria for prioritising any ideas that I proposed in this paper – ‘thinking’ resembles the ‘feasibility/rational/operational’, ‘feeling’ is the basis of the ‘attractiveness/emotional/subjective’, while ‘willing’ resembles the ‘impact on life over time through action’.

Perception is the ‘organization, identification, and interpretation of sensory information in order to represent and understand the environment’ [21]. All perception arises from signals in the nervous system, which are triggered by physical or chemical stimulation of the sense organs. While perception relies on intricate nervous system processes, it feels largely effortless because much of this processing occurs outside conscious awareness. Some philosophers argue that the goal of perception is to gain knowledge, but evolutionary psychologists emphasise its primary role in guiding action. In the concept proposed in this paper, perception of ideas and their assessment and prioritisation would, in fact, guide action (‘pursuing ideas’); however, the prioritisation would need to be based on a good understanding of the context, which is acquired through perception from other senses, all of which are integrated in the brain. So, my proposed concept of the brain’s ‘sense of ideas’ shows that both of the previous views would be correct, to an extent.

Since developing and maintaining organs for major senses demands significant energy, senses will evolve only when they substantially enhance an organism's chances for survival. Sensory processing occupies more than half of the brain, which itself uses about a quarter of the body's metabolic resources, highlighting the critical role of the senses in improving fitness of living beings [21]. It is likely that the sense of ‘perception of ideas’ using the brain should improve fitness, as surviving must have been historically associated with generating and prioritising better ideas. Therefore, the speed of brain’s evolution in human body should be faster than for other organs, especially for the genes involved in the structure and function of this ‘sense’. Interestingly, it is now possible to rank regions in the human genome that show significant evolutionary acceleration. The most dramatic acceleration has been noticed in so-called ‘human accelerated regions’ (HAR), in a novel RNA gene (HAR1F). This gene is expressed specifically in Cajal-Retzius neurons in the developing human neocortex from 7 to 19 gestational weeks, which is a crucial period for cortical neuron specification and migration [22,23]. It would be interesting to explore whether mutations in this gene affect the process of prioritising ideas.

The term ‘senses’ can be generally understood as the physiological abilities of organisms to gather data for perception. More precisely, a sense can be defined as ‘a system comprising a group of sensory cell types that respond to a particular physical phenomenon, linked to specific brain regions where the signals are processed and interpreted.’ Aristotle is credited with identifying the traditional ‘five senses’—sight, hearing, touch, smell, and taste—based on the primary sensory organs, as detailed in his *De Anima* (‘On the Soul’) [24]. It was not until two thousand years later that the vestibular organ's role as a sensor of ‘balance and movement’ was identified, earning Austrian scientist Robert Barany the Nobel Prize in 1914 for his research on ‘the physiology and pathology of the vestibular apparatus’. Modern science has since acknowledged the existence of additional human ‘senses’ supported by specialized neuronal receptors, though they lack distinct organs. This has sparked debates among physiologists and psychologists over how to categorise and quantify human senses, with proposals ranging from nine to more than twenty [24].

These debates may be somewhat misplaced, as it is likely that the sensory organs of modern humans evolved from simple clusters of progressively specialised neuronal receptors before developing into distinct organs. Furthermore, the sensory organs continued to evolve, giving rise to several ‘sub-senses’ within the broader ‘major’ senses. For instance, some sensory organs develop diverse, increasingly specialised types of neuronal cells to enhance the quality of sensory data. Examples include the eye's two distinct light-sensitive cells – rods and cones – or the tongue's taste buds, which can detect several different flavours. These complexities further complicate efforts to classify human senses [24]. In any case, there still seem to be opportunities for new senses to be added to the list based on novel insights.

All the information collected by sensory organs and neuronal receptors is continuously integrated and processed by the brain. Interestingly, as mentioned before, despite its central role, the brain itself is not commonly recognised in Western culture or mainstream scientific literature as an organ associated with any specific sense. In contrast, Buddhist philosophy includes the mind as a ‘sense-base’ (*Ayatana*), alongside the traditional five senses. This perspective likely stems from the psychological focus inherent in Buddhist practices and teachings. The traditional five senses are referred to as the ‘five material faculties’ and they appear in symbolic form as early as the *Katha Upanishad* (approximately 6th century BCE), depicted as five horses pulling the body's ‘chariot,’ with the mind acting as the ‘charioteer’. Similarly, Tamil literature, including the *Tolkappiyam*, identifies the mind as a distinct sense organ, describing beings as possessing six senses, with the mind complementing the traditional five. One verse states, ‘The beings with six senses have a mind, along with the above’ [24].

If the brain’s perception of ideas, as a distinct human sense, is to be better understood through the ‘science of ideas’, measurement tools and techniques will need to be devised that will allow testing various hypotheses. The book ‘Measuring Ideas: The CHNRI Method’ explains how ideas can be measured through quantifying collective optimism of experts towards each idea’s potential to satisfy pre-defined criteria that can discriminate between the many proposed ideas [9]. It is noteworthy to mention that, in the mid-20th century, social scientists began exploring how and why ideas spread between individuals and across cultures. Everett M. Rogers was a pioneer in this field, conducting research on the ‘diffusion of innovations’ and identifying factors influencing idea adoption, as well as the profiles of adopters [25]. Later, in 1976, Richard Dawkins proposed applying evolutionary biology concepts to the spread of ideas in his book ‘The Selfish Gene’ [26]. He introduced the term ‘meme’ to describe an abstract unit of cultural transmission, analogous to the gene in biological evolution. In 2000, Michael E. Porter and Scott Stern studied patenting activity across countries as a way to ‘measure the potential in human ideas’, which is an exciting approach to this problem from the field of economics [27].

## CONCLUSIONS AND FUTURE STEPS

The proposed view of the brain as the sensor of the ideas could lead to many quantitative or qualitative experiments to further explore the properties of this ‘sense’ in both individuals and populations, establishing the ‘science of ideas’, based on the proposed additional human sense – the ‘brain’s perception of ideas’. Ideas are offering a vision of the future different from the most probable one, if the idea is followed through with action using the required energy and effort. Ideas will generate excitement and enthusiasm in some people, and scepticism and fear in others. Experiments could be designed to explore how is the ‘sense of ideas’ associated to the level of education, experience, cognitive abilities, personality type, and whether there is indeed a ‘normal’ and ‘impaired’ response to being exposed to an idea. The brain’s perception of ideas may be an important sense to recognise, because knowing to tell a good idea from a bad one is certainly helpful for survival and it should provide evolutionary advantage. The rise of humans as a species, their rapid understanding of the nature, the universe, and the environment that they live in, and their influence of the planet Earth might be partially influenced by acquiring the sense of perceiving the ideas and getting enthused, motivated, and energised by them in their individual and collective actions.
